# Rational Design of Stapled Covalent Peptide Modifiers of Oncoprotein E6 from Human Papillomavirus

**DOI:** 10.1021/acschembio.4c00878

**Published:** 2025-03-10

**Authors:** Cole Emanuelson, Yuta Naro, Olivia Shade, Melinda Liu, Sagar D. Khare, Alexander Deiters

**Affiliations:** † Department of Chemistry, University of Pittsburgh, Pittsburgh, Pennsylvania 15260, United States; ‡ Department of Chemistry and Chemical Biology, Rutgers University, Piscataway, New Jersey 08854, United States

## Abstract

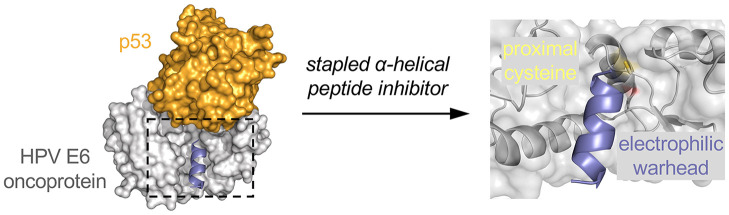

Human Papillomavirus (HPV) is linked to multiple cancers, most significantly cervical cancer, for which HPV infection is associated with nearly all cases. Essential to the oncogenesis of HPV is the function of the viral protein E6 and its role in degrading the cell cycle regulator p53. Degradation of p53, and the resultant loss of cell cycle control, is mediated by E6 recruitment of the E3 ubiquitin ligase E6AP and subsequent ubiquitination of p53. Here, we report the design of a stapled peptide that mimics the LxxLL α-helical domain of E6AP to bind and covalently label a cysteine residue specific to HPV-16 E6. Several acrylamide- and haloacetamide-based warheads were evaluated for reactivity and specificity, and a panel of hydrocarbon-stapled peptides was evaluated for enhanced binding affinity and increased proteolytic stability. Structure-based modeling was used to rationalize the observed trends in the reactivity of the warheads and the impact of the hydrocarbon staple position on the binding affinity of the stapled peptides. The development of a proteolytically stable and reactive peptide represents a new class of peptide-based inhibitors of protein–protein interactions with a potential therapeutic value toward HPV-derived cancers.

## Introduction

Human Papillomavirus (HPV) is a double stranded DNA virus with a genome of approximately 8 kb encoding eight genes.^1^ High-risk HPV is an established carcinogen in cervical, vaginal, penile, and oropharyngeal cancers and can be attributed to 5% of all cancers worldwide.^2^ There are 13 genotypes of HPV considered high-risk; however, the most common are HPV-16 and HPV-18.^3^ In particular, HPV-16 poses the highest risk of cancer development, with a majority of cervical and noncervical HPV-related cancers being attributed to HPV-16.^2^

Two viral proteins, E6 and E7, are the main drivers of oncogenesis.^4−6^ To access the host cell’s replication machinery, the E6 and E7 oncoproteins drive the cell into S phase by targeting vital pathways that enable replicative immortality, evade growth suppressors, promote genome instability, and resist cell death.^7^ E7 function is largely attributed to its ability to bind and degrade the cell cycle regulators pRb, p107, p105, and p130.^8^ E6 functions by hijacking the endogenous E3 ubiquitin ligase E6AP, leading to proteasomal degradation of the tumor suppressor protein p53.^7^ Loss of p53 inhibits apoptosis and deregulates DNA damage response mechanisms in infected cells.^9−11^ As such, the essential role of HPV-16 E6 presents its interaction with endogenous E6AP as a potential therapeutic target.

While nucleic acid-based approaches such as RNA interference^12−14^ and antisense agents^15,16^ have been developed with the ability to reduce E6 protein levels and stabilize p53 levels in cells, challenges with cellular stability, delivery, and off-target effects remain as significant limitations.^17,18^ Several small molecule inhibitors, identified through high-throughput screens, were found to disrupt the E6-E6AP interaction in vitro and inhibit E6-mediated degradation of p53 in HPV positive cells; however, these compounds had poor potency and did not effectively induce apoptosis in HPV-infected cells.^19^ A significant challenge to targeting E6 with small molecule inhibitors is the inherent absence of a defined ligand binding pocket, rendering E6 “undruggable” to traditional approaches, as the ability to disrupt protein–protein interactions (PPI) across a large surface area is notoriously difficult to achieve using small molecule ligands.^20^ Unlike small molecules, larger peptides can adeptly probe the binding interface of PPIs and the design of peptide-based inhibitors can be aided by structural characterization of the binding interface and the specific arrangement of the constituent peptide motifs. Viral hijacking of E6AP via E6 is guided by a short leucine-rich LxxLL, where x denotes any amino acid, α-helix consensus motif located near the C-terminus of E6AP.^21^ Peptide inhibitors derived from the natural LxxLL motif have demonstrated the ability to competitively inhibit the E6/E6AP interaction in vitro^22,23^ and are capable of rescuing p53 protein levels in HPV-positive cells when introduced through a peptide-expressing plasmid.^24,25^ Additional evidence of p53 rescue by peptide inhibitors of E6 have exploited a separate mechanism of E6 that relies on the inhibition of the p53 coactivators p300 and CBP, which acetylates p53 increasing its stability and activity.^26^ Expression of the CH1 domain of p300 in HPV-positive HNSCC cells was found to increase total p53 and enhance transcriptional activity.^27^

Development of peptide-based inhibitors of PPIs that rely on classical competitive inhibition can be challenging due to the high affinity of PPIs as a result of their large contact surfaces, which in the case of E6-E6AP has been determined to be 139 nM.^28^ Relative to ligands that reversibly engage their target, covalent inhibitors exhibit enhanced potency and selectivity and prolonged duration of action.^29−32^ Here, we report the rational design of covalent peptide ligands for HPV-16 E6 through the introduction of acrylamide and haloacetamide electrophiles. Furthermore, the basic E6AP LxxLL motif was modified to introduce a rigidifying hydrocarbon staple into the backbone, conferring enhanced binding affinity and proteolytic stability. The results presented here demonstrate a path toward effective peptide-based inhibitors for HPV-16 E6.

## Results and Discussion

**Figure 1:**
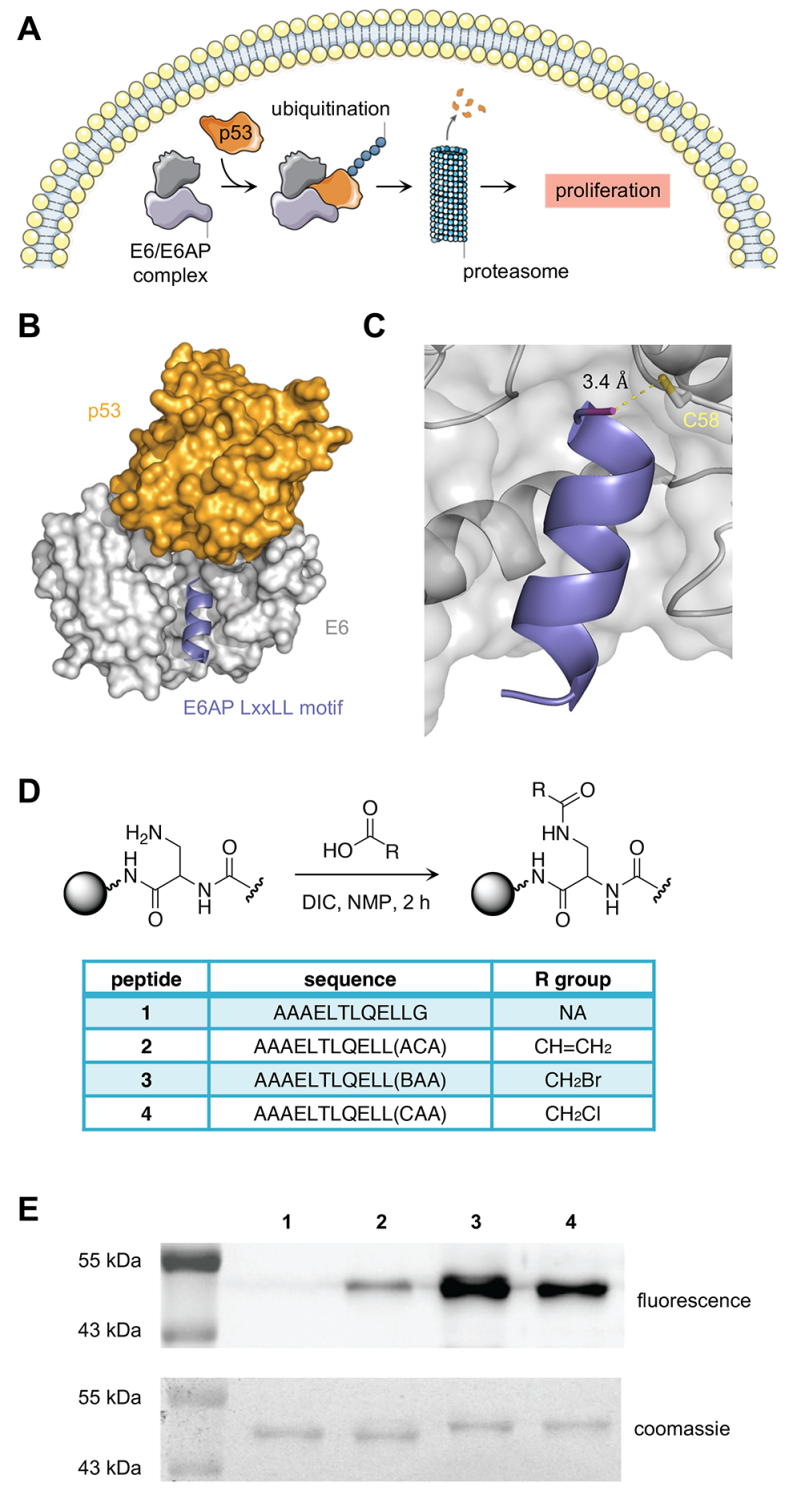
(A) Role of oncoprotein E6 in oncogenesis. E6 (gray) forms a complex with the endogenous ubiquitin ligase E6AP (purple), and this complex recruits the tumor suppressor protein p53 (orange) forming an E6/E6AP/p53 complex that facilitates degradation of p53 through the ubiquitin-proteasome pathway. (B) Ternary complex of full length HPV-16 E6 (gray), the core domain of p53 (orange), and the LxxLL α-helical motif of E6AP (purple) prepared from a crystal structure of the ternary complex (PDB: 4XR8). (C) Binding pocket cysteine residue C58 is located 3.4 Å from the terminal glycine residue. (D) Electrophilic warhead installation by solid phase peptide syntheses. (E) Gel shift analysis of covalent labeling of GST-E6 protein by in-gel fluorescence and coomassie stain after 30 min incubation.

To design a potent HPV-16 E6 inhibitor, the core LxxLL α-helix motif was investigated as a scaffold for the development of a covalent ligand. Given the importance of the role of E6AP in promoting HPV-16 E6-mediated degradation of p53, the development of a covalent peptide inhibitor of E6 would serve as an initial step toward a new treatment approach for HPV-positive cancers (Figure 1A). Inspection of the crystal structure of E6 bound to the LxxLL motif of E6AP led to the recognition of a cysteine residue (C58) within the E6AP:E6 interface, 3.4 Å from the C-terminal glycine of the LxxLL α-helical motif (Figure 1B,C; PDB: 4XR8). Importantly, modification of the motif at this position places an electrophile within a distance amenable to covalent bond formation. This is supported by computational investigations of covalently targeted cysteine residues, which included both acrylamide and haloacetamide warheads that showed that the mean distance between the electrophilic warhead and the thiol group of labeled cysteines is less than 10 Å.^33^ While literature precedence exists for acrylamide and haloacetamide electrophile targeting of cysteine, experimental demonstration of labeling is necessary to confirm that the specific orientation of the modified peptide within the E6AP:E6 binding interface supports the distinct 1,4-addition of acrylamides, and the nucleophilic substitution of the halogen by haloacetamides.^34^

To provide a site for electrophile installation, the C-terminal glycine of the E6AP LxxLL α-helical motif was substituted with 2,3-diaminopropanoic acid (Dap), which can be readily derivatized before cleavage of the peptide from the solid-support. The deprotected Dap was modified with carboxylic acid derivatives using diisoproylcarbodiimide (DIC) coupling to install acrylamide, bromoacetamide, and chloroacetamide electrophiles (Figure 1D). These electrophiles were chosen for their known cysteine reactivity and range in electrophilicity,^35^ as well as their established use with modified peptides for the covalent labeling of cysteines on proteins.^36^ These previous studies present a consistent ranking of electrophile reactivity toward cysteine: acrylamide < chloroacetamide < bromoacetamide. To monitor binding interactions, a fluorophore was installed at the amino terminus of each peptide through conjugation to 5-carboxyfluorescein. All peptides were purified by reverse phase HPLC and characterized by MALDI-MS (Figure S1 and Table S1).

Glutathione S-transferase (GST) tagged HPV-16 E6 (47 kDa) was expressed in *E. coli*, purified (Figure S2), and incubated at a final concentration of 1 μM with 1.25 eq of unmodified peptide **1**, acrylamide **2**, bromoacetamide **3**, or chloroacetamide **4** for 30 min at 37 °C. To experimentally assess covalent labeling of E6, a gel shift assay under denaturing conditions was conducted, as successful covalent bond formation should result in the addition of ∼1.7 kDa to the mass of the protein and enable separation based on differential gel retention. Using in-gel fluorescence to detect labeling of E6 by the fluorescently tagged peptides, we observed a fluorescent band at the expected molecular weight for each covalent peptide (Figure 1E). Coomassie staining of the SDS-PAGE gel was used to visualize unlabeled E6 and assess the extent of labeling by each peptide. Treatment with **3** and **4** resulted in complete labeling, while only a faint band with a higher molecular weight was observed for the less electrophilic **2**.

**Figure 2:**
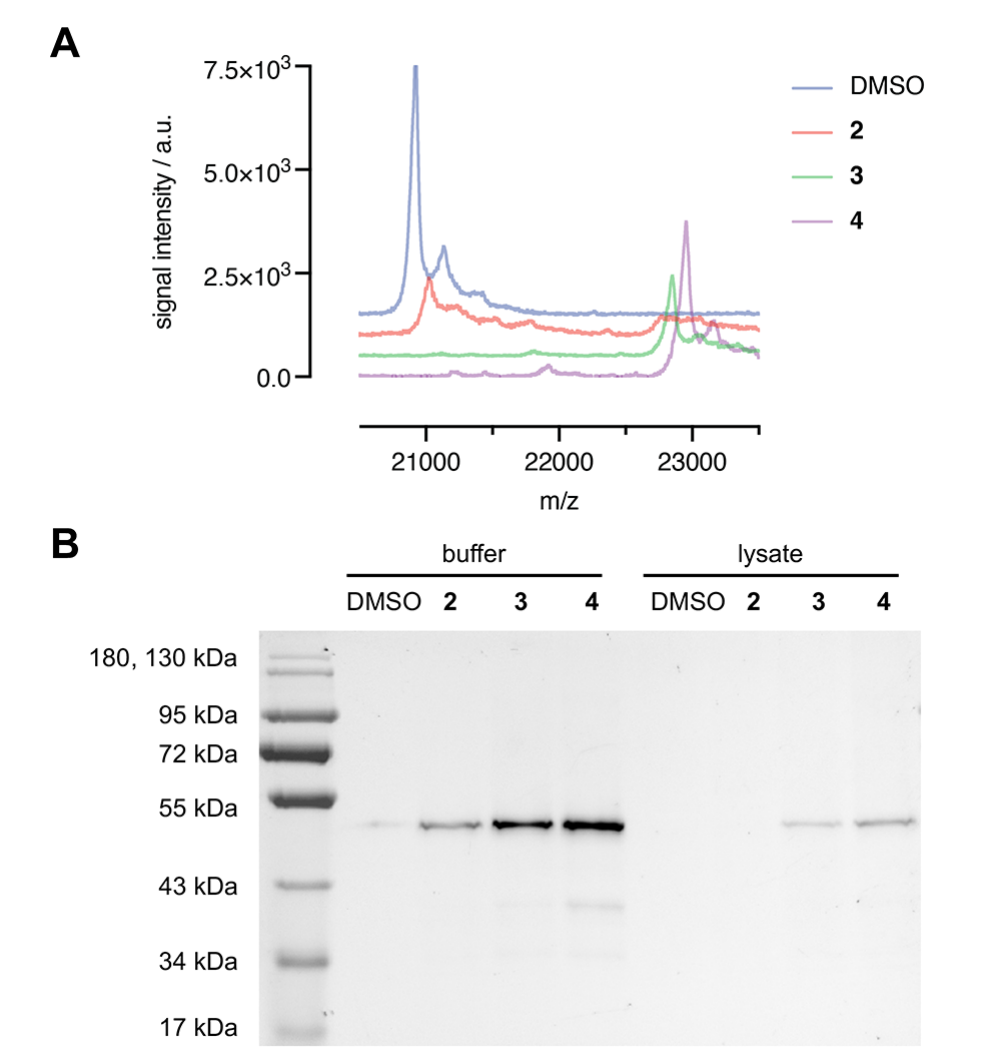
(A) MALDI-MS spectra of recombinant E6 protein following 1 h incubation with covalent peptides **2**−**4** or DMSO. (B) GST-E6 was incubated with **2**, **3**, or **4** (1.25 equiv) with and without whole cell HEK293T lysate for 30 min. Samples were separated by SDS-PAGE and visualized using in-gel fluorescence.

To further confirm the E6 labeling, mass spectrometry analyses were performed. Recombinant E6 was first subjected to thrombin cleavage to remove the GST affinity tag, followed by MALDI-MS analysis of a mixture of E6 and covalent peptides. Upon incubation with DMSO alone, the GST-cleaved protein was observed at a mass of 20.9 kDa. Covalent labeling resulted in the addition of 1.7 kDa through cross-linking of the peptide, which was clearly detected in the mass spectrum. Consistent with the gel shift analysis, the greatest amount of labeling was observed for **3** and **4,** while only a limited degree of labeling was found for **2** (Figure 2A).

To further probe the reactivity of these warheads, a labeling time course was conducted with each peptide, and the full-length GST-E6 protein and reactions were assessed using MALDI-MS (Figure S3). Labeling of the full-length protein was observed as before, with the bromo- and chloroacetamide peptides **3** and **4** showing complete labeling by 15 and 30 min, respectively. The acrylamide showed limited labeling at each time point, with a mass signal for the addition adduct that was not readily detectable over the background. While similar acrylamide warheads have been utilized in the context of cysteine-directed peptide inhibitors, high efficiency target labeling was only reported when excess inhibitor was used.^36,37^

Next, the selectivity of covalent peptides **2**–**4** was assessed by carrying out the in vitro labeling assay in the presence of off-target proteins. GST-E6 was incubated with **2**–**4** (1.25 equiv) in both Tris buffer and HEK293T lysate at 37 °C for 30 min (Figure 2B). In-gel fluorescence of the reaction mixtures suggests that incubation in cell lysate results in a reduction in labeling efficiency for each warhead, but most significantly for **3**, which shows a substantial decrease in band intensity. In the case of **2**, no detectable amount of labeling was seen. Further, covalent peptides **2**–**4** (1.25 equiv) were incubated with recombinant GST (1 equiv) in Tris buffer to determine off-target labeling (Figure S4). Peptides **2** and **4** displayed minimal labeling of GST, whereas peptide **3** did react with GST, indicating the increased promiscuity of the electrophile.

**Figure 3:**
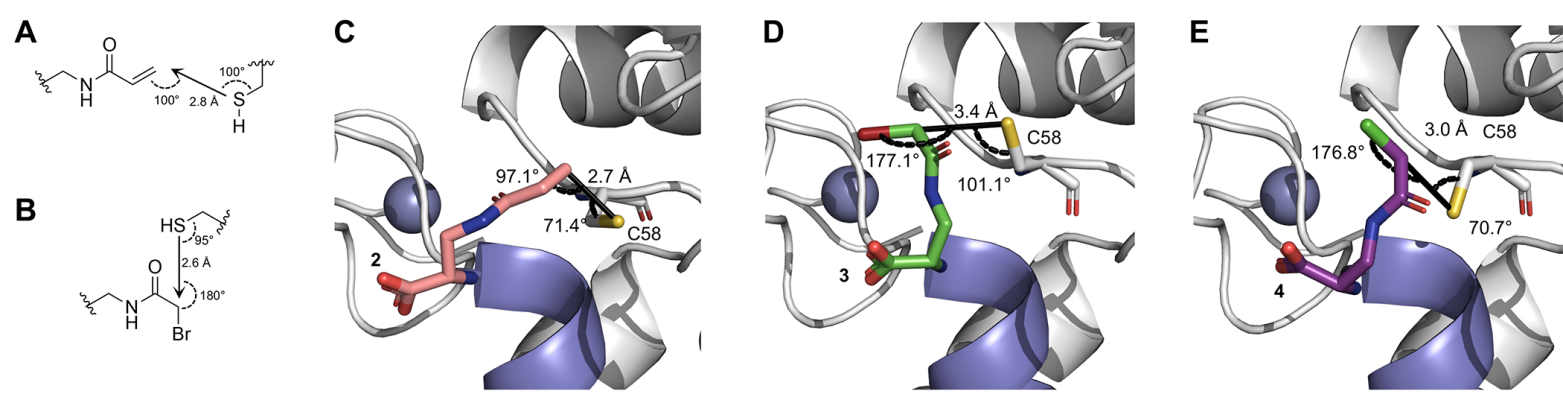
(A) 1,4-Addition transition state with QM/MM-generated attack geometry.^39^ (B) Scheme of the S_N_2 substitution transition state with QM-generated attack geometry.^38^ (C) Peptide **2** ACA staple forming rotamer (salmon). (D) Representative peptide **3** BAA staple forming rotamer (green). (E) Example peptide **4** CAA staple forming rotamer (purple). Attack angles and distances are labeled and shown in black dashes.

To investigate the reactivity of the acrylamide- and haloacetamide-modified peptides, computational modeling was performed with Rosetta. Each reactive warhead was modeled as a C-terminal unnatural amino acid and evaluated for its ability to react with the target cysteine. Relative positioning of the warhead rotamers with respect to the target cysteine were compared to reaction geometries required for covalent bond formation using established mechanisms—1,4-addition for acrylamide (Figure 3A) and S_N_2 nucleophilic substitution for haloacetamides (Figure 3B).^38,39^ The electrophilic side chains contain four dihedrals, with the haloacetamides having 24 plausible rotamers each and the acrylamide having 8 plausible rotamers. As such, the haloacetamides inherently have more flexibility than the acrylamide.

In the context of the bound structures and considering all allowed (backbone-dependent) rotamers for the reactive Cys side chain, only one acrylamide rotamer was in the position to react (Figure 3C), whereas five to seven haloacetamide rotamers (Figure 3D,E) were in the position for nucleophilic substitution (Figure S5 and Tables S2–S4), indicating that the covalent cross-linking is more favorable with haloacetamide than acrylamide warheads. The greater leaving group propensity of the bromine vs chlorine is not considered in Rosetta models, thus precluding any quantitative comparisons between the two haloacetamides using this approach. Overall, our analysis shows that the haloacetamide warheads on the C-terminal residue have a greater number of energetically favorable and cross-linking-compatible conformations than the acrylamide warhead. This analysis aligns with the observed higher reactivity with bromo- and chloroacetamides than with acrylamide (Figure 1E).

**Figure 4:**
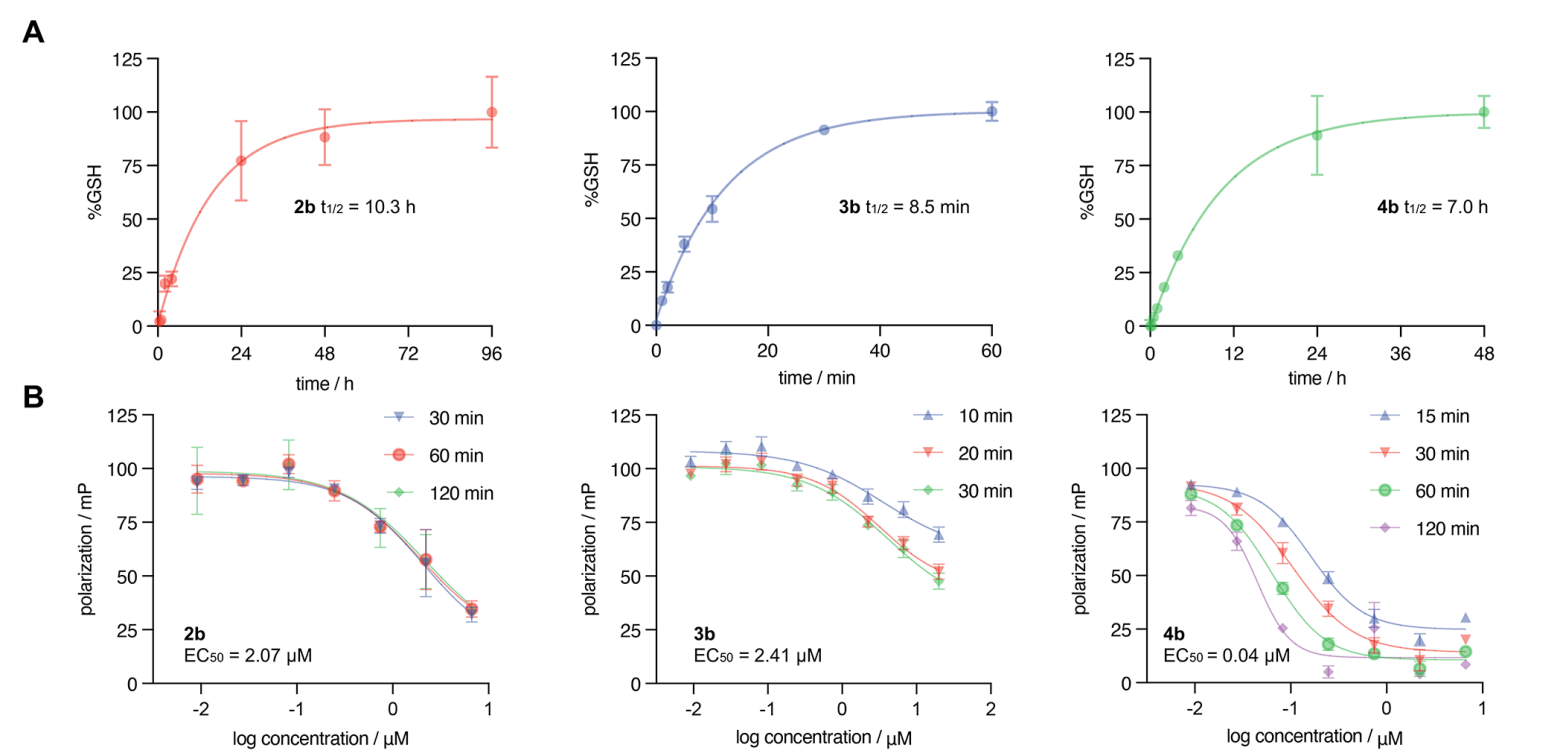
(A) Determination of glutathione reactivity of covalent warheads by HPLC. The percent GSH adduct formed, as determined by the measured product peak area, is plotted versus reaction time. Data represent the mean normalized peak area for GSH adduct (±SD, *n* = 3), and nonlinear fit was applied using GraphPad Prism. (B) Competitive fluorescence polarization (FP) of the fluorescent E6 peptide ligand with GST-E6 and varying concentrations of covalent peptide. Data represent the mean polarization (±SD, *n* = 3) and was fitted to a sigmoidal dose–response curve using GraphPad Prism. EC_50_ values were determined to be 2.07 μM (90% CI of 1.02 to 64.1 μM) for **2b**, 2.41 μM (95% CI of 1.35 to 10.1 μM) for **3b**, and 0.0491 μM (95% CI of 0.00897 to 0.0786 μM) for **4b**.

Having confirmed and rationalized the labeling of E6 by **2**, **3**, and **4**, we next sought to better characterize the reactivity of each warhead to determine the best choice for further probe development and optimization. To evaluate the reactivity toward off-target thiols, the acrylamide, bromoacetamide, and chloroacetamide covalent peptides were exposed to glutathione (10 equiv) in an HPLC time course assay over 96 h (Figure 4A). Here, a set of nonfluorescent peptides (**2b**, **3b**, and **4b**) were synthesized with an N-terminal tryptophan for quantification through absorbance measurements. Glutathione is the most abundant thiol in human cells,^40^ and time point measurements were obtained by quenching reactions with cold acetonitrile containing TFA (0.1%) and 5-carboxyfluorescein as an internal standard. Each warhead showed a propensity to react with free thiols, and the resulting half-lives showed trends consistent with electrophilicity: **2b***t*_1/2_ = 10.3 h, **3b***t*_1/2_ = 8.5 min, and **4b***t*_1/2_ = 7.0 h. In the context of small molecules, similar *t*_1/2_ values were reported for glutathione reactivity of these warheads, suggesting that the significantly different structural context does not alter off-target reactivity and that the chloroacetamide **4** is preferred over the bromoacetamide **3**.^35^

Next, covalent inhibition was evaluated by using a competitive FP assay. Recombinant GST-E6 was pre-incubated with a fluorescently labeled ligand for 30 min before addition of varying concentrations of the non-fluorescent covalent peptides (Figure 4B). Fluorophore polarization is greatest when the ligand is bound to its target, and consequently, a displacement of the ligand by the covalent peptide results in a decrease in polarization.^41,42^ The formation of a covalent bond between the peptide inhibitor and the C58 residue within E6 is expected to occur more slowly than noncovalent binding, permitting characterization of the affinity of the peptides **2b**, **3b**, and **4b**.^41^ Indeed, the observed effective concentration of chloroacetamide **4b** (0.24–0.04 μM) decreased over time, whereas the EC_50_ values for **2b** (2.07 μM) and **3b** (2.41 μM) remained relatively static over the course of the assay. The change in potency observed for **4b** is consistent with the expected outcome for a covalent inhibitor, as covalent bond formation reduced the fraction of free E6 and resulted in a shift of the binding curve. It is possible that acrylamide **2b** reacts too slowly to produce an observable change under these experimental conditions. Given its rapid reaction with glutathione, bromoacetamide **3b** may be consumed by thiols in the reaction buffer, which includes 1 mM DTT. A test of off-target reactivity of **3b** with DTT (1 mM) showed a half-life of only 2.1 min (Figure S6), supporting the observed instability in DTT-containing buffer. Importantly, these covalent peptide inhibitors effectively compete for the E6AP binding site of E6, which is a key feature in this potential therapeutic approach. Having demonstrated successful covalent engagement of E6 using different in vitro binding assays, additional strategies to further improve the efficacy of chloroacetamide modified inhibitor **4** were considered.

To successfully block HPV-16 E6, the peptide ligand must be taken up by oncogene expressing cells; adequate cellular concentrations need to be achieved, and E6AP needs to be displaced. Unfortunately, most peptide ligands comprised primarily of natural amino acid residues, similar to **4**, suffer from limited “drug-like” properties, including limited binding affinity and poor proteolytic stability. Peptide stapling stabilizes the α-helical conformation of peptides by forming a bridge between residues across one or more α-helical turns and has the potential to address both of these limitations.^43^ Short α-helical peptide ligands, such as the E6AP LxxLL α-helical motif, exhibit high conformational instability and often exist in a dynamic equilibrium between a transient α-helical structure and a random coil state in solution.^44,45^ The stabilization of the helical conformation imparted by backbone stapling reduces the entropic penalty upon peptide binding, resulting in significantly enhanced binding affinity.^46^ Furthermore, the unnatural bridge improves proteolytic stability of peptides by reducing susceptibility to proteases.^47^

**Figure 5:**
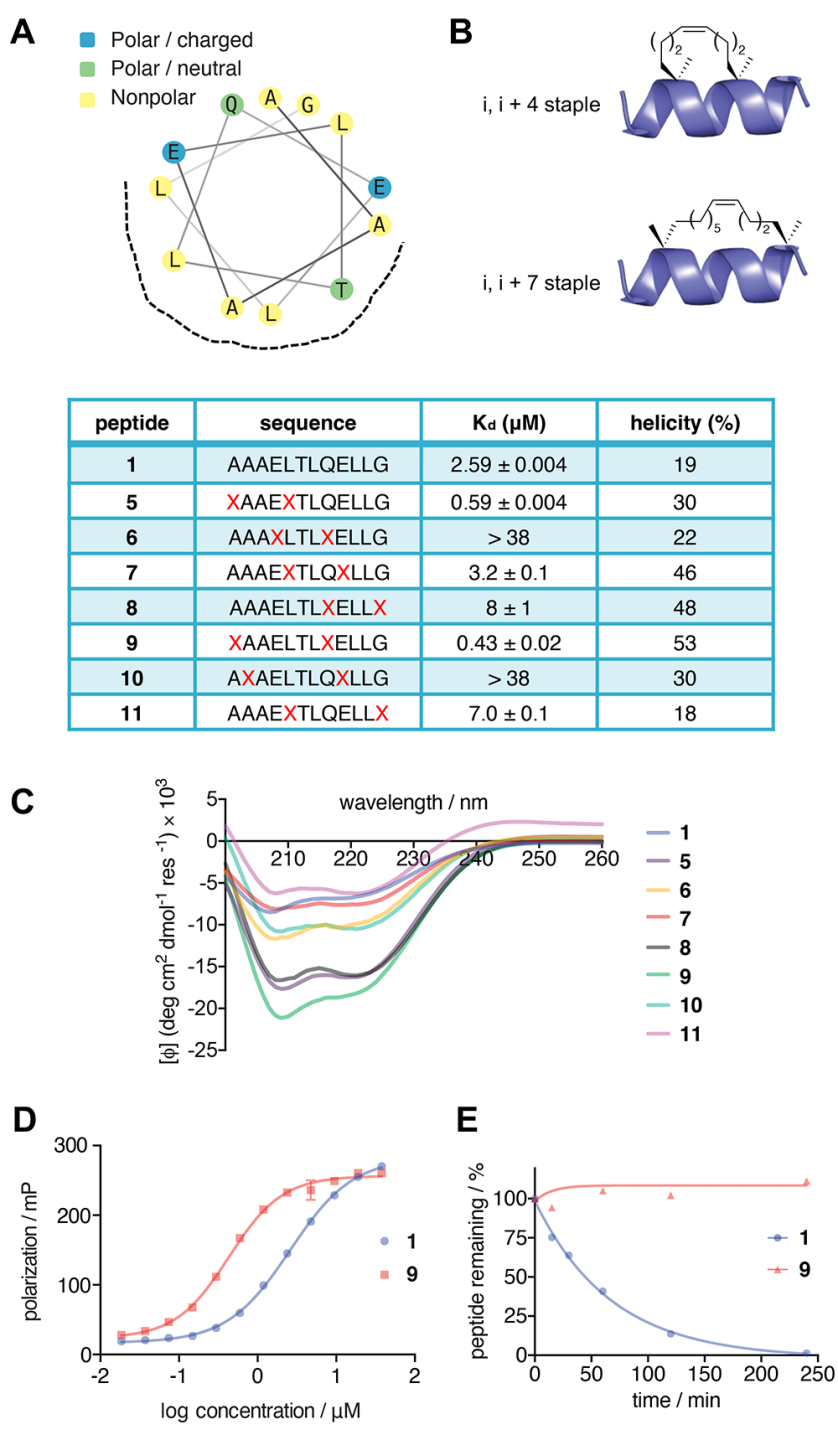
(A) Helical-wheel representation of the LxxLL motif of E6AP, wherein the binding surface of E6 is represented by a dashed line. (B) Hydrocarbon staple formation is accomplished through introduction of α-methyl, α-alkenyl amino acids at positions (*i*, *i* + 4, top) or (*i*, *i* + 7, bottom) followed by on-resin ring-closing olefin metathesis. (C) Circular dichroism (CD) spectrum of stapled peptide derivatives. Percent helicity was determined using the molar ellipticity at 222 nm. (D) Binding affinity (*K*_D_) of stapled peptide derivatives with recombinant E6 protein was determined by FP. Data represent the mean polarization (±SD, *n* = 2) and were fitted to a sigmoidal dose–response curve using GraphPad Prism. EC_50_ values calculated for **1** and **9** were determined to be 2.59 μM (95% CI of 2.43 to 2.76 μM) and 0.431 μM (95% CI of 0.374 to 0.493 μM), respectively. (E) Peptide stability upon treatment with chymotrypsin as determined by HPLC analysis. The percent intact peptide was measured by quantification of the peak area.

To rationally design a covalent, stapled peptide targeting HPV-16 E6, the crystal structure of the E6AP peptide bound to E6 was used to generate a helical wheel diagram revealing both the solvent-exposed residues and the E6-E6AP interface of the peptide (Figure 5A). Using this diagram as a model, solvent exposed residues and residues residing at the border of the E6-E6AP interface were chosen for incorporation of hydrocarbon staples. α-Methyl, α,α-disubstituted amino acids were employed as hydrocarbon staples due to their well-documented success across a variety of substrates and their synthetic accessibility.^46,48^ Introduction of these staples is accomplished by the incorporation of olefin-containing amino acids followed by an intramolecular ring-closing metathesis (RCM) using ruthenium-based Grubbs catalysts.

To this end, a panel of (*i*, *i* + 4) and (*i*, *i* + 7, Figure 5B) stapled peptides (**5–11**) was synthesized using microwave-assisted Fmoc SPPS and RCM.^47^ An N-terminal carboxyfluorescein was included for FP analysis of binding affinity for both the natural backbone and the stapled peptides. The peptide **1** exhibited a *K*_D_ of 2.59 μM toward E6-GST, matching literature reports.^24,49^ Peptides **6**, **7**, and **8** containing (*i*, *i* + 4) staples showed reduced affinity (*K*_D_ ≥ 38, 3, and 8 μM, respectively, Figure 5B), which may be due to the disruption of the hydrogen bond interactions of the substituted glutamic acid residues or steric clash of the staple at the E6 binding interface.^21^ Introduction of (*i*, *i* + 7) staples in peptides **10** and **11** also showed significant losses in binding affinity, with *K*_D_ of >38 and 7 μM, respectively (Figure 5B). Peptide **10**, similar to **7**, has been substituted at a glutamic acid residue that forms stabilizing polar interactions with E6, while **11** has been substituted at the terminal glycine residue, which was similarly poorly tolerated in **8**. Peptide **5,** containing an (*i*, *i* + 4) staple at the N-terminus, exhibited a promising 4.4-fold improvement in binding affinity (*K*_D_ = 0.59 μM) compared to wild-type peptide **1** (*K*_D_ = 2.59 μM). The (*i*, *i* + 7) stapled peptide **9** showed a 6-fold enhancement in binding affinity (*K*_D_ = 0.43 μM) relative to that of **1**. Significantly, neither peptides **5** nor **9** were stapled at the glutamic acid or glycine residues within the binding motif, highlighting the importance of preserving the stabilizing interactions of these residues. The biophysical and stability properties of the two enhanced peptide ligands, **5** and **9**, were then investigated.

To determine the secondary structure of peptides **1**–**11**, CD was measured in an aqueous solution. All the peptides exhibited a distinct spectral profile associated with helical secondary structure, indicated by the double minima at 208 and 222 nm.^50^ The molar ellipticity ([θ]) at 222 nm was used to estimate the percent helicity of peptides in aqueous solutions and enable direct comparison of the helical content of peptides **1–11** (Figure 5C).^51,52^ While the wild-type unstapled peptide **1** exhibited only 20% helical content, most of the stapled peptides showed significantly enhanced helical contents, with values ranging between 24%–56% helicity for peptides **5–11**. Peptide **9**, which showed the largest enhancement in affinity, had the most pronounced helicity (56%), supporting that α-helix stabilization can enhance ligand affinity. The peptides **7** and **8** which showed reduced binding affinity were found to also have enhanced helical content, demonstrating the importance of assessing the contributions of both the secondary structure and stabilizing side-chain interactions to the overall binding affinity.

To determine whether hydrocarbon staple introduction would also enhance proteolytic stability of the E6AP peptide, the wild-type peptide **1** and the stapled peptide **9**, which showed the greatest enhancement in binding affinity (Figure 5D), were incubated with chymotrypsin, and degradation was monitored via HPLC.^53^ Chymotrypsin was chosen due to its substrate selectivity for peptides containing hydrophobic side chains, including leucine, making the LxxLL motif a well-suited substrate for proteolytic cleavage by this protease.^54^ While complete degradation of **1** was observed within 4 h (*t*_1/2_ = 47 min), no degradation of **9** was observed in the same time (Figure 5E). Continued incubation for up to 16 h led to no significant degradation of peptide **9**, clearly demonstrating an improvement in proteolytic stability through stapling.

**Figure 6:**
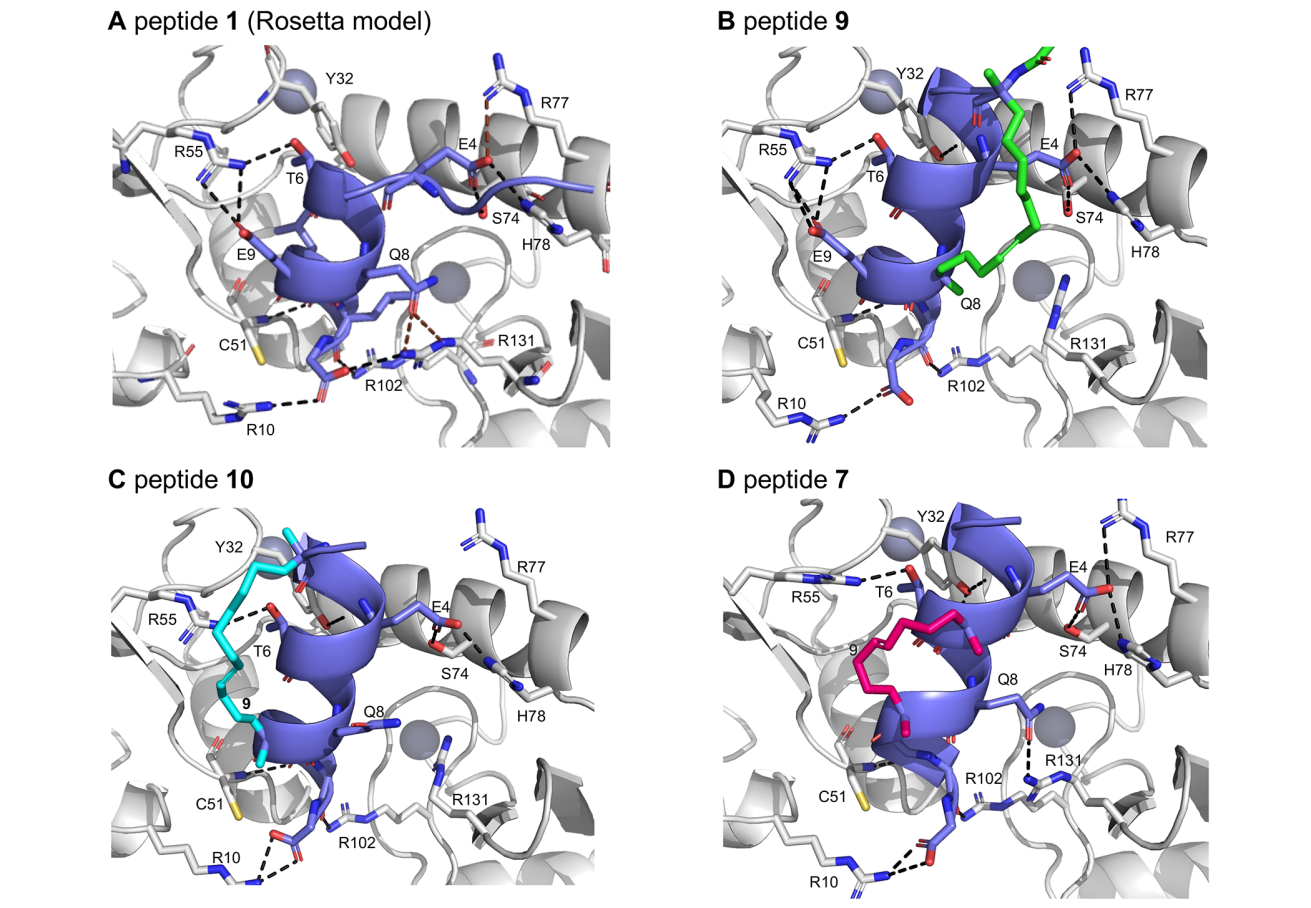
Models of bound complexes. (A) Peptide **1** with LxxLL motif in purple, truncated from E6AP structure and after Rosetta energy minimization. E6 is shown in gray (PDB 4XR8). Polar interactions in black are referred to as core interactions from the crystal structure, whereas polar interactions in brown, between E4-R77 and Q8-R131, are additional interactions revealed by Rosetta. Models of peptides (B) **9**, (C) **6**, and (D) **10**. Dashed black lines refer to polar contacts between E4, T6, Q8, E9 of the peptides, and R10, Y32, C51, R55, S74, R77, H78, R102, and R131 of E6.

To rationalize the effect of the staple on binding affinity, computational modeling was performed with Rosetta and Amber. Examination of the E6/E6AP crystal structure revealed a set of polar interactions between the side chains (Glu4, Thr6, Glu9) and the main chain atom (Leu10, Leu11, and Gly12) groups of the peptide helix with residues in the E6 protein (Arg10, Cys51, Arg55, Ser74, His78, and Arg102, Figure S7A). Rosetta energy minimization of the crystal structure revealed the possibility of additional supporting interactions through helix-Glu4 with E6-Arg77 and helix-Gln8 with E6-Arg131 (Figure 6A). The helicity induced due to the staple also placed the helix backbone in a position to hydrogen bond with Tyr32 of E6 (Figure 6B–D). These key interactions are maintained in the models of peptides **5** and **9** (Figures 6B and S7B, respectively). The hydrocarbon staple enforced greater local helicity in the interaction hotspot, which is consistent with the improved binding affinity of **5** and **9** (*K*_D_ < 1 μM) compared to the wild-type **1** (*K*_D_ = 2.59 μM, Figure 5A).

Substitution of key glutamic acid residues Glu4 and Glu9 disrupted the interactions of peptides **6** and **10** (Figures 6C and S7C, respectively) with E6, which is consistent with their experimentally observed low affinity (*K*_D_ > 38 μM; Figure 5). Similar to peptide **10**, substitution of key residue Glu9 in peptide **7** abolishes interchain interactions with E6. However, the conformation and positioning of peptide **7** form the auxiliary Glu4-Arg77 and Gln8-Arg131 interactions and allow for the backbone O and N atoms of residues Ala3 and Leu7, respectively, to form two new hydrogen bonds with Tyr32 of E6 (Figure S8). These interactions may restore some of the binding affinity (*K*_D_ = 3.2 μM; Figure 6D).

Peptides **8** and **11** yielded *K*_D_ values that are 3–4-fold weaker than those of wild-type **1** and are stapled via the C-terminal glycine. Our models suggest that this modification introduces distortions within the peptide helix, leading to the loss of main chain hydrogen bonds between Glu4-Gln8 and Leu7-Leu11 (Figure S9). While some of the interchain binding interactions can still be formed, the required strain in the helix may be responsible for the loss of binding affinity (Figure S7D,E). Overall, the modeling of the stapled peptides qualitatively recapitulates the observed trends in binding affinity and shows that the location of staples can affect the stability of the binding-competent conformation as well as the network of polar interactions with E6.

**Figure 7:**
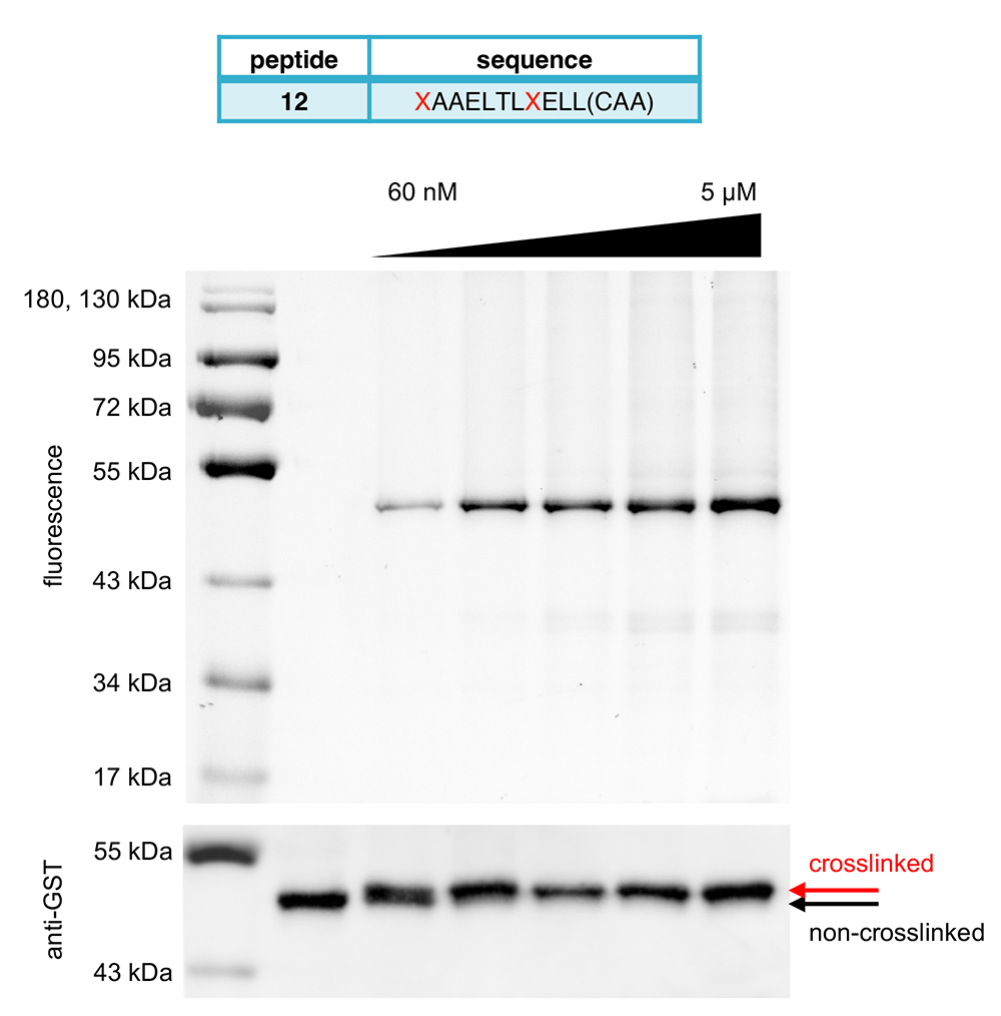
Recombinant GST-E6 (1 μM) labeled with increasing concentrations of **12**. Covalent peptide **12** in a 3-fold serial dilution (treatment concentrations were 5, 1.67, 0.56, 0.19, and 0.06 μM) or DMSO was added to a mixture of GST-E6 in cell lysate then incubated for 30 min. In-gel fluorescence was used to detect covalent labeling of the fluorescent peptide, and a Western blot was completed using an anti-GST antibody to assess the extent of labeling.

Having evaluated the properties of candidate electrophiles and completed an SAR study of staple modifications leading to peptides with enhanced binding affinity and stability, we selected peptide **9** to serve as a scaffold for the installation of the chloroacetamide electrophile. This electrophile showed improved selectivity and potency relative to those of the acrylamide and bromoacetamide warheads. Peptide **12** was synthesized with an (*i, i* + 7) hydrocarbon staple by SPPS and RCM, as described above, and a chloroacetamide, installed through substitution of the C-terminal glycine in **9** with Dap and on-resin coupling with chloroacetic acid. To evaluate the reactivity and selectivity of **12** for E6, a labeling assay was conducted by incubation of GST-E6 (1 μM) with increasing amounts of **12** (60 nM to 5 μM) in whole cell HEK293T lysate (Figure 7). Covalent modification was observed by an in-gel fluorescence analysis of the reaction mixture. Importantly, while a decrease in labeling efficiency in lysate was seen for peptides **2**–**4**, which lack backbone modification (Figure 2B), **12** was observed to efficiently label GST-E6 protein in whole cell lysate, with observable labeling at concentrations as low as 60 nM via in-gel fluorescence. At higher concentrations of **12** (5–1.67 μM), complete labeling of GST-E6 protein was observed as determined by Western blot detection of band migration using an anti-GST antibody. This is enabled by covalent bond formation with the helical peptide, which results in an addition of ∼1.8 kDa and a corresponding gel shift. The improved selectivity and labeling efficiency of **12** relative to the peptides with native backbones may be attributed to the enhanced binding affinity and proteolytic resistance imparted by the stapled α-helix.

## Conclusions

In summary, using a rational design approach to mimic the scaffold of a native protein–protein interaction (PPI) interface, a stapled peptide was engineered to be a covalent inhibitor of the HPV-16 protein E6. While appreciation for the utility of covalent inhibitors is increasing, concern over off-target labeling and potential biological assay interference has historically limited their advancement.^29,31,55^ Prediction of the optimal warhead that balances on- and off-target reactivity is complicated by context-dependent influences on overall reactivity, including ligand and target conformation.^56^ However, covalent inhibitors possess enhanced potency due to increased residence time and a prolonged duration of action. This advantage makes them particularly applicable in the development of PPI inhibitors, which are characterized by large flat interaction surfaces that are difficult to probe with traditional small molecule binders that must out-compete endogenous protein ligands.^56^ The PPI between HPV-16 viral protein E6 and endogenous E3 ubiquitin ligase E6AP represents one such binding interface with potential therapeutic relevance that is difficult to disrupt using small molecules. The E6-E6AP interaction is key to the HPV pathology, as binding results in proteasomal degradation of the tumor suppressor protein p53 and an inhibition of apoptosis and deregulation of DNA damage response mechanisms in expressing cells.^7,9−11^

Here, we identified a nucleophilic cysteine residue in the proximity of the peptide–protein interface of E6-E6AP and tested a small panel of electrophiles for their capacity to covalently engage the E6 target protein when installed on a mimic of this motif. It was observed that a bromoacetamide electrophile that exhibited relatively fast reactivity with glutathione in solution showed poor selectivity for target protein labeling in vitro. Instead, slower-reacting chloroacetamide displayed improved on-target selectivity. Backbone modification of short α-helical peptides using linkages such as hydrocarbon staples has been shown to be an effective method for increasing drug-like properties of bioactive peptides by increasing proteolytic resistance and binding affinity.^43,57^ Through systematic stapling of the natural α-helical LxxLL recognition motif, we discovered that introduction of an (*i*, *i* + 7) hydrocarbon staple produced a >6-fold improvement in binding affinity and a substantial enhancement in proteolytic stability.

Structure-based computational models were used to rationalize the observed trends in warhead reactivity and the impact of stapling on the binding affinity. These analyses indicate that in the context of the E6 protein–peptide interface structure, haloacetamides are more reactive due to the greater number of accessible conformations compatible with covalent cross-linking to the target cysteine compared to acrylamide. Furthermore, models show how covalent stapling locations that lead to higher affinity maintain key interchain interaction networks and increase the helicity of the peptide, leading to higher stability of the bound conformation compared with the unstapled peptide. Accurate computation-based recapitulation of experimentally observed trends in reactivity and binding may aid in the future design of covalent peptides with high reactivity and affinity.

Based on these insights, a stapled peptide bearing a chloroacetamide warhead was synthesized and demonstrated enhanced labeling efficiency and selectivity toward E6 in whole cell lysate. Stapled, covalent cysteine-targeting peptides have shown promising activity in cells and recently in a mouse A549 xenograft animal model. Luo et al. demonstrated that a covalent peptide targeted to C360 of the histone demethylase LSD1 was capable of effectively inhibiting tumor growth.^58^ Our studies showcase that this approach is viable for the disruption of oncogenic PPIs. However, continued work is necessary for the development of stapled covalent inhibitors of HPV-16 E6 including an investigation of the cellular response to inhibition and potential to rescue p53 activity. Taken together, this study demonstrates a potential approach toward the development of HPV-targeted therapeutics and diagnostics.

## Materials and Methods

### General Chemical Information

All of the reagents and solvents were purchased from commercial suppliers and used without further purification. Reverse phase HPLC was carried out on a Shimadzu Prominence HPLC system. Products were eluded using 30 min gradients between water (0.1% TFA, solvent A) and acetonitrile (0.1% TFA, solvent B), monitored by UV detection at 220 and 280 nm. HPLC purification was carried out using an Agilent SB-C18 semiprep column with a flow rate of 4 mL/min, 100–400 μL injection volume, and an automated fraction collector. Analytical HPLC was carried out using SB-C18 analytical column with a flow rate of 2 mL/min and 10–20 μL injection volume. MALDI-TOF mass spectrum analysis was performed on a Bruker ultrafleXtreme using α-cyano-4-hydroxycinnamic acid and sinapinic acid as an ionization matrix. Unless noted otherwise, the final DMSO content within assays was maintained at 0.5% [v/v].

### Peptide Synthesis

All peptides were synthesized using microwave-assisted Fmoc solid phase methods on a CEM Discover system using NovaPEG rink amide resin (EMD Millipore) or Rink Amide ChemMatrix (Biotage, 7-600-1310) PEG-based polystyrene resin, with an average loading between 0.4 and 0.7 mmol/g, corresponding to 125 mg (50 μmol linker) and 71 mg (50 μmol linker) of dried resin, respectively. Synthesis was carried out at 50 μmol scales in a 10 mL fritted syringe open reaction vessel (Torqiv) with a micro magnetic stir bar. Microwave reactions were carried out in a CEM Discovery reactor with an open-vessel adaptor, open-vessel mode selected, and stirring set to high. A fiber optic probe was used to measure reaction temperature, and coupling and deprotection programs were set to reach target temperature over 1 min with a maximum power of 75 W, followed by incubation for the remaining reaction time with a maximum power of 15 W. Stock solution of coupling reagent, 0.2 M *N*,*N*′-diisopropylcarbodiimide (DIC) and 0.5 M ethyl cyano(hydroxylimino)acetate (Oxyma) in *N*-methyl-2-pyrrolidone (NMP), was prepared for *n* coupling reactions by adding *n* × 31 μL of DIC and *n* × 71 mg of Oxyma to *n* × 1 mL of NMP. Fmoc-protected amino acids (4.0 eq, 0.2 mmol) were pre-weighed into 1.5 mL Eppendorf tubes. Immediately prior to addition to the reaction syringe, the aliquots were preactivated by addition and dissolution with 1 mL of coupling reagent. Coupling of standard Fmoc-protected amino acids were performed at 90 °C for 2 min. Coupling reactions of Fmoc-(*S*)-2-(4-pentenyl)Ala-OH (4 eq, 0.2 mmol, 76 mg) and Fmoc-(*R*)-2-(7-octenyl)Ala-OH (4 eq, 0.2 mmol, 84 mg) were performed at 90 °C for 4 min. Coupling reactions of 5-carboxylfluorescein (4 equiv, 0.2 mmol, 75 mg), activated as described above, were performed at 50 °C for 30 min. The N-terminus of peptides terminating in tryptophan was acetylated on resin with acetic anhydride (10 eq, 0.5 mmol, 47 μL) and *N*,*N*-diisopropylethylamine (50 eq, 2.5 mmol, 435 μL) in NMP (1.6 mL) at RT with stirring for 30 min. Fmoc deprotections were performed with 20% (v/v) 4-methylpiperidine in dimethylformamide (2 mL) at 90 °C for 1 min. Resin washes (3 × 1 min) were performed between the coupling and deprotection steps using dimethylformamide (3 mL). All peptide sequences include an N-terminal β-alanine spacing residue. RCM reactions (2 × 120 min) were performed on-resin using Grubbs Catalyst first Generation (0.2 eq, 0.01 mmol, 8.23 mg) in dichloroethane (1 mL) at RT. Covalent peptides were synthesized with the incorporation of N_β_-Mtt-N_α_-Fmoc-2,3-diaminopropionic acid, followed by selective side chain deprotection with 1% [v/v] trifluoroactic acid (TFA) and 2% [v/v] triisopropyl silane (TIPS) in DCM (2 mL) for 30 min. Coupling to the deprotected side chain was then carried out by addition of carboxylic acid (5 eq, 0.25 mmol; bromoacetic acid, 35 mg; chloroacetic acid, 24 mg; or acrylic acid, 17 μL) preactivated as described above with DIC (5 eq, 0.25 mmol, 39 μL) in 1 mL of NMP, followed by stirring at RT for 2 h. Final peptides were cleaved from the resin and fully deprotected by treatment with cleavage cocktail (1 mL; 95% [v/v] TFA, 2.5% water, 2.5% [v/v] TIPS) at room temp for 3 h. Cleaved peptides were precipitated into ice-cold ether (25 mL) and centrifuged (10 min at 10,000 g) to a pellet. The pellet was resuspended in acetonitrile:water (1:1), filtered, and then purified by reverse phase semipreparative HPLC.^59^ Purified peptide fractions were frozen at −80 °C for 2 h and lyophilized to dryness overnight (Labconco Freezone 6). Lyophilized peptides were dissolved in DMSO (100 μL) and stored at −20 °C. To determine peptide stock concentration, peptide DMSO stock was diluted 1:1000 in 10 mM phosphate buffer (1 mL; pH 8.2) in a quartz cuvette, and absorbance was measured (Tecan M200) at 494 nm (fluorescein-labeled peptides) or 280 nm (tryptophan-containing peptides). The molar extinction coefficients used for concentration determination were 82,000 cm^–1^ M^–1^ and 5500 cm^–^1 M^–1^ for fluorescein and tryptophan containing peptides, respectively, and typical stock concentrations were 5–20 mM.^59^

### Recombinant Expression of GST-Tagged HPV16 E6 Protein

A bacterial expression construct containing the GST-tagged HPV16 E6 gene (GST-HPV16 E6, Addgene #24127)^60^ was transformed into competent BL21 *E. coli,* and a single colony was picked for generation of a 10 mL starter culture in LB broth with ampicillin (100 μg/mL) and incubated overnight in a 37 °C shaker. The following day, the 10 mL starter culture was used to inoculate 1 L of LB broth culture containing ampicillin (100 μg/mL) and was then incubated in a 37 °C shaker while monitoring the OD600 at 30 min intervals using a Tecan M200 cuvette reader and an uninoculated LB broth aliquot as a blank. At OD600 = 0.5 (typically reached after 3–4 h), isopropyl β-d-1-thiogalactopyranoside (1 mL of a 1 M stock in water) was added to a final concentration of 1 mM, and the culture was moved to a 17 °C shaker and incubated for an additional 24 h. Cells were pelleted by centrifugation (20 min, 5000*g*, 4 °C) and then resuspended in 50 mL of lysis buffer (50 mM Tris, 400 mM NaCl, 5 mM DTT, 1% Triton X-100, and 1 mg mL^–1^ lysozyme, pH = 8) and incubated on ice for 1 h. Cells were lysed by sonication (15% power, 10 min; 20 s on and 20 s off, FisherScientific 550 Sonic Dismembrator); to reduce viscosity, DNase I was added (10 units, 10 μL of 1 U/μL stock in water) and incubated on ice for 10 min. Debris was pelleted by centrifugation (20 min, 18,000*g*, 4 °C). Supernatant containing protein was added to 1 mL of glutathione agarose resin (50% [v/v] slurry Pierce) and incubated overnight at 4 °C with gentle shaking. Resin was pelleted (5 min, 1000 g), supernatant was removed, and resin was washed once with lysis buffer (10 mL, 15 min) and then washed twice with GST wash buffer (10 mL; 50 mM Tris, 400 mM NaCl, 5 mM DTT, pH = 7.5). Protein was eluted from resin using elution buffer (6 × 500 μL; 50 mM Tris, 400 mM NaCl, 10 mM reduced glutathione, 5 mM DTT, 0.1% Trition X-100), and elution fractions were concentrated by spin filtration with Amicon Ultra 30K MW column (10 min, 14,000 g). To remove excess glutathione, elution buffer without glutathione (500 μL) was added to the column and centrifuged (10 min, 14,000*g*) a total of five times to yield a concentrated protein stock (∼100 μL). Concentrated protein stocks were divided into ∼10 μL aliquots to limit freeze–thaw cycles and stored at −80 °C until use. To determine the concentration of protein stock, an SDS-PAGE gel containing BSA standards (5 μL of 2.0, 1.0, and 0.5 mg mL^–1^ solutions) and a sample of protein stock (5 μL) was run (60 V, 15 min; 250 V, 1 h), stained with Coomassie, imaged, and quantified using BioRad Image Lab software and densitometry analysis using a calibration curve fitted to the BSA standards.

### In Vitro E6 Labeling Assays

GST-E6 (1 μM) was incubated with peptides (1.25 equiv; from 10× working stock in buffer with 5% [v/v] DMSO content) for 30 min at 37 °C in buffer (to 50 μL total volume; 50 mM Tris, 200 mM NaCl, 1 mM DTT, 0.1% [v/v] Trition X-100) with a final DMSO content of 0.5% [v/v]. Labeling reaction mixtures were separated by gel electrophoresis on 16% SDS-PAGE. Detection of fluorescent labeling was measured by in gel fluorescence using a GFP filter, and total protein was detected by Coomassie staining. Images were obtained using a ChemiDoc MP Imaging System.

### In Vitro E6 Labeling Assays in Cell Lysate

HEK293T cells were maintained in Dulbecco’s Modified Eagle Medium supplemented with 10% (v/v) fetal bovine serum (Sigma-Aldrich, F0926) and 1% (v/v) penicillin/streptomycin (Corning, 30002CI) at 37 °C with 5% CO_2_ in a 10 cm plate. Cells were detached with 1 mL of TrypLE enzyme (Gibco, 12604013), counted by hemocytometer, pelleted by centrifugation (500*g*, 5 min), and washed with PBS (2 × 1 mL). The PBS-washed cell pellet was lysed on ice in RIPA buffer (Thermo Fisher, 89,900) at a concentration of 1 × 10^6^ cells/mL. GST-E6 (1 μM) was incubated with peptides (1.25 equiv or indicated concentration; from a 10× working stock in buffer with 5% [v/v] DMSO content) in whole cell lysate (to 50 μL total volume with a final DMSO content of 0.5% [v/v]) at 37 °C for 30 min. Labeling reactions were separated by gel electrophoresis on 16% SDS-PAGE (60 V, 15 min; 250 V, 1 h), and detection of covalent labeling was assessed by in gel fluorescence using a GFP filter, followed by Western blot. Proteins were transferred (80 V, 1.5 h) to a PVDF membrane (GE Healthcare), and the membrane was incubated in blocking buffer [10 mL, 5% BSA in TBS with 0.1% [v/v] Tween 20 (TBST)] for 1 h at RT. The blots were probed with primary anti-GST antibody (1:10,000 dilution, Proteintech, 10,000-0-AP) in TBST (10 mL) overnight at 4 °C with rocking. Blots were then washed with TBST (3 × 10 mL, 10 min) at RT. Next, blots were incubated with antirabbit HRP conjugate secondary antibodies (1:10,000 dilution, Proteintech, SA00001-2) for 1 h at RT with rocking, followed by washing with TBST (3 × 10 mL, 10 min) at RT. Chemiluminescence was developed using a SuperSignal West Pico Chemiluminescent Substrate (ThermoFisher, 8 mL) and imaged on a ChemiDoc MP Imaging System using automatic exposure settings.

### Fluorescence Polarization Binding Assays

For noncompetitive FP experiments, varying concentrations of GST-E6 protein (38 μM; 10 point 2-fold serial dilution) were incubated with 30 nM fluorescently labeled peptides in buffer (10 μL total volume; 50 mM Tris, 200 mM NaCl, 1 mM DTT, 0.1% [v/v] Trition X-100) in black 384-well plates. For competitive FP experiments, 100 nM GST-E6 was incubated with 30 nM fluorescently labeled noncovalent peptide and varying concentrations (20 μM; 3-fold serial dilution) of covalent peptide in buffer (20 μL) with a final DMSO content of 0.5% [v/v]. Negative control (polarization reference; 30 nM fluorescent peptide, no protein) and blank reference (buffer alone) were included in triplicate for *G*-factor calibration. Sample plates were centrifuged (1 min, 500*g*) and incubated for 30 min at RT. Following incubation, FP at equilibrium was measured on a Tecan M1000 microplate reader (ex = 470 nm, em = 525; gain optimized, 200 flashes, 300 ms settle time, *G*-factor reference value 30 mP). Measured polarizations were fitted to a sigmoidal dose–response curve using GraphPad Prism software to generate *K*_D_ values.

### Glutathione Reactivity Assay

Covalent peptides (100 μM) were diluted from 10 mM DMSO stock solutions (1% final DMSO content) and incubated in buffer (500 μL total volume; 50 mM Tris, 200 mM NaCl) with glutathione (10 equiv, 1 mM) at 37 °C. To quench the reaction, 10 μL of the reaction mixture was added to 100 μL of cold acetonitrile containing 0.1% TFA [v/v] and 10 μM fluorescein, included as an internal standard. Reaction mixtures were first analyzed by LCMS after 1 h of incubation to assign the glutathione adduct peak by comparison to mixtures quenched immediately following peptide addition. For each time point, from 0 min to 96 h, quenched samples were analyzed in triplicate by reverse phase analytical HPLC using a gradient of 5–95% acetonitrile (0.1% TFA) in water (0.1% TFA), a flow rate of 2 mL min^–1^, and a detection wavelength of 280 nm to quantify the area under the curve (AUC) of the resulting GSH adduct peak. The AUC of the reacted covalent peptide was normalized to the AUC of the fluorescein standard. These values were plotted and fitted with nonlinear regression using GraphPad Prism software.

### In Vitro Proteolytic Stability Assays

For proteolytic testing, peptides (5 μL) were diluted from 5 mM DMSO stock solutions into proteolysis buffer (500 μL; 50 mM Tris, 150 mM NaCl, pH = 7.5) containing bovine α-chymotrypsin (50 nM) to give a final concentration of (50 μM, 1% DMSO) and incubated at room temp. For each time point, a 50 μL aliquot was removed from the reaction mixture and quenched with 40 μL of 0.5% [v/v] trifluoroacetic acid in acetonitrile. Quenched samples were subsequently analyzed by analytical reverse-phase HPLC, and the remaining undigested peptide was quantified by integration of AUC. A plot of percent peptide remaining vs time was fit to a one-phase exponential decay to calculate half-life using GraphPad Prism software.

### Circular Dichroism Spectroscopy

CD measurements were acquired on an Olis DSM17 CD spectrophotometer. Cells with a path length of 2 mm were used. Peptide solutions at 50 μM were prepared in 10 mM phosphate buffer (500 μL; pH = 7.2) containing 0.5% of DMSO, and exact concentrations were determined using fluorescein absorbance at 494 nm and a molar extinction coefficient of 82,000 cm^–1^ M^–1^.^59^ Blank solutions of buffer without peptide were prepared for background subtraction. Scans were performed at 20 °C from 200 to 260 nm with increments of 1 nm, bandwidth of 2 nm, and a 5 s integration time. Ellipticity (θ) was normalized to the peptide concentration and to the number of residues to yield normalized ellipticity [θ]. Normalized ellipticity was plotted using GraphPad Prism and values, and curves were smoothed using a smoothing function without differentiating or integrating and using 8 neighbors on each side and a second order smoothing polynomial.^61^ Helical content for peptides was calculated using a previously described method^52^ that assumes that the peptide population only exists in two states, helical and random coil, and that the ellipticity contribution from the random coil population is negligible at 222 nm. The percent helical content was estimated by dividing the observed ellipticity at 222 nm, [θ_obs_]_222_, in deg cm^2^ dmol^–1^ by the limiting ellipticity value for a 100% helical peptide. This was calculated via the equation [θ_obs_]_222_ = 43,000(1 – [*x*/*n*]), where *x* is the factor accounting for end effects, for which 2.5 was used, and *n* is the number of residues.^52^

### Computational Modeling

Computational models were generated using PyRosetta, a macromolecular modeling suite, and AMBER20, a molecular dynamics simulation package.^62,63^ Chemical structures of the electrophilic and stapling unnatural amino acids were created with Avogadro.^64^ To determine possible rotamer dihedral values for each unnatural, each dihedral was searched with the Mogul tool from Cambridge Crystallographic Data Centre (CCDC), which references structures from the Cambridge Structural Database (CSD), to find common values for similar dihedrals in existing structures.^65^ Those with less than 100 reference structures in the CSD were further scanned using the HF/6-31g(d) basis set in Gaussian to find the values between 0° and 360° that correspond to energy minima of the unnatural.^66^ The stapling unnatural amino acids and their staple geometries were further parametrized using the protein ff19SB force field in Amber to define our altered amino acids and modify the force fields to accommodate our unconventional system.^67^ The structure of the E6/E6AP complex was from the Protein Databank (PDB) file accession code 4XR8, where the LxxLL peptide was truncated from the larger E6AP protein.^21^ Rosetta energies were calculated with the beta_nov16 score function, with constraints on the E6/LxxLL peptide polar interaction geometry to model a bound complex.^68^ The complex underwent Rosetta FastRelax for initial minimization.^63^

For investigating the covalent binding to E6, the C-terminal glycine of the LxxLL helix was substituted into each rotamer for each of the electrophilic unnatural amino acids. The rotamer libraries for all of the unnaturals were generated by combinatorial sampling of all possible values for all dihedrals. FastRelax was performed on residues within 8 Å to sample for energetically feasible conformations in context of the electrophile.^63^ The geometry of each rotamer and target cysteine was compared to the 1,4 addition geometry, for the acrylamide warhead, and to the nucleophilic substitution geometry, for the haloacetamide warhead.^38,39^ Rotamers with attack distances within 0.7 A and angles within 30° were accepted as reaction-compatible.

For investigating the helical stapling within the LxxLL peptide, RFdiffusion and Rosetta were used to generate a full ideal helical peptide to model the stabilizing effects of the staple. Relevant residues were then mutated into the corresponding unnatural amino acids with Rosetta to generate starting structures for each stapled peptide. A double bond was introduced between the unnaturals to generate the staple, with double bond geometries enforced using additional geometric constraints in PyRosetta and additional force field modifications in Amber. To obtain accurate double bond geometries for the staple in our models, Amber energy minimizations and molecular dynamic simulations were run on the Rosetta mutated peptides, generating unbound structures of each peptide in context of the staple in a neutralized 10 A explicit solvent box of OPC water, to match the ff19SB force field used with the peptide.^67^ The resulting equilibrated peptides at the end of the production runs, whose RMSDs from their initial structures varied from 0.85 to 1.7 A, were docked in position with respect to the E6 in PyMol, based on the LxxLL motif placement in PDB 4XR8, before FastRelax was performed on the entire helix and neighboring residues on E6 within 8 Å of the helix. 25 trajectories were generated per peptide, and the models with the lowest energies were chosen for comparisons. Polar contacts were determined by using PyMol.

## Supporting Information Available

Additional characterization of peptides including HPLC chromatograms and MALDI-MS spectra, plasmid map and SDS-PAGE of recombinant E6 protein expression and purification, SDS-PAGE of covalent labeling, calculated structures of electrophilic warhead rotamers, models of peptide–protein interactions, and tables of calculated angles and cysteine-warhead distances (PDF)
