# Supplementary material for: Rational Design of Stapled Covalent Peptide Modifiers of Oncoprotein E6 from Human Papillomavirus
Source: ACS Chem Biol. 2025 Mar 10;20(3):746–57. doi: 10.1021/acschembio.4c00878 (PMC11934087; doi:10.1021/acschembio.4c00878)
Supplement: Supplementary file 1 — cb4c00878_si_001.pdf [file cb4c00878_si_001.pdf]

Supporting Information

Rational Design of Stapled Covalent Peptide Modifiers of Oncoprotein E6 from Human Papillomavirus

Cole Emanuelson, Yuta Naro, Olivia Shade, Melinda Liu, Sagar D. Khare, and Alexander Deiters*

University of Pittsburgh, Department of Chemistry, Pittsburgh, PA 15260

Rutgers University, Department of Chemistry and Chemical Biology, Piscataway, NJ 08854

**To whom correspondence should be addressed: deiters@pitt.edu*

Supporting Figures & Tables

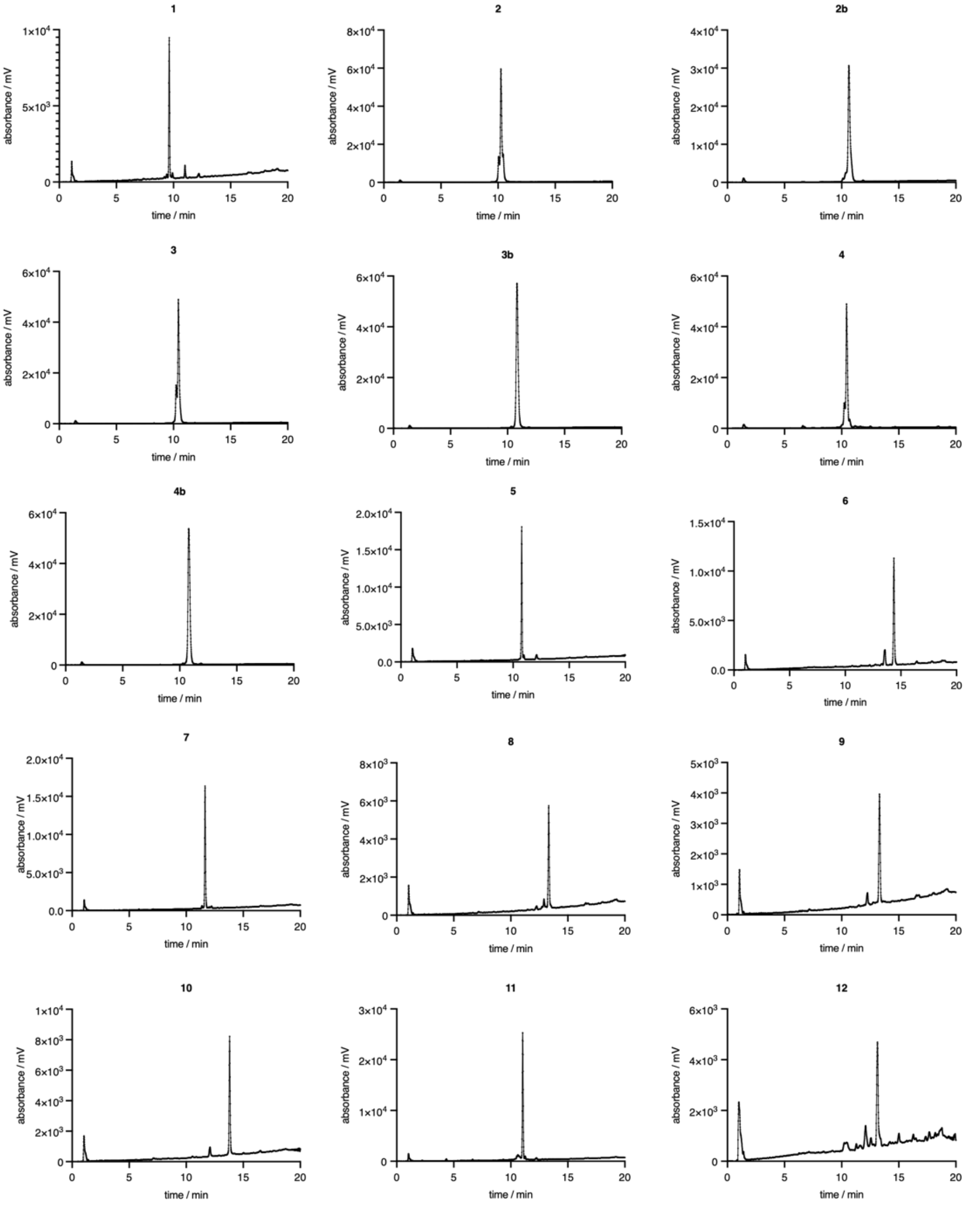

peptide	1	2	2b	3	3b	4	4b	5	6	7	8	9	10	11	12
purity (%)	99	99	98	100	99	100	100	100	100	100	97	97	96	98	95

Supporting Figure S1. Analytical HPLC chromatograms for purified peptides **2-4**. Peptides were eluted using a gradient of 5–95% acetonitrile (0.1% TFA) in water (0.1% TFA), a flow rate of 2 mL/min, and detection wavelength of 280 nm. The DMSO cosolvent elutes at 1 min. Percent purity of each peptide was calculated via area under the curve using Prism 10.

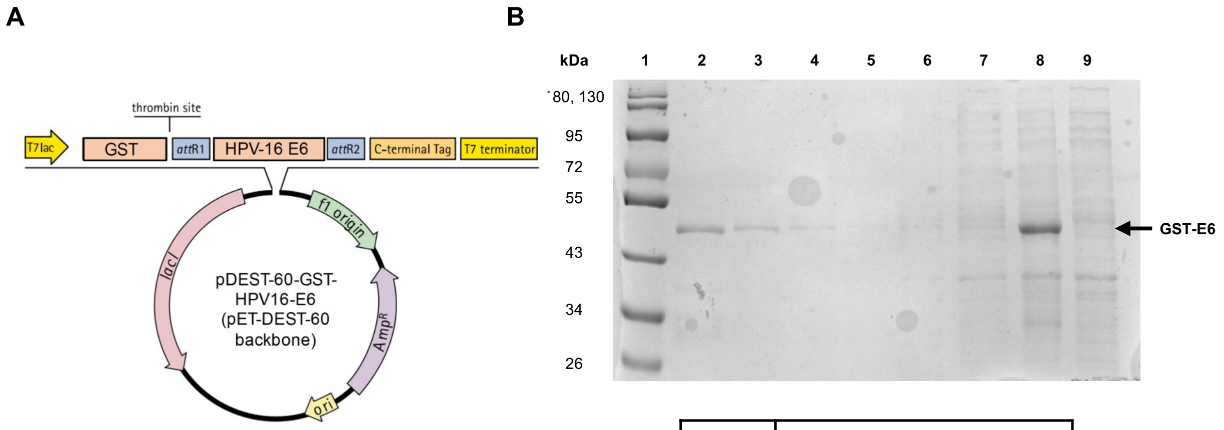

lane	sample
1	protein marker ladder
2-5	purification elution fractions
6	wash fraction
7	glutathione resin supernatant
8	whole cell pellet +IPTG culture
9	whole cell pellet –IPTG culture

Supporting Figure S2. GST-E6 expression and characterization. A) Plasmid map for HPV16 GST-E6-fusion protein expression (Addgene #24127). B) GST-E6 was purified from transformed B21 *E. coli* using glutathione agarose resin. Elution fractions from purification (lanes 2-5) were used to assess purity. Minimal GST-E6 protein was eluted during resin washing (Lane 6) or remaining in crude lysate after glutathione agarose purification (Lane 7). As expected, analysis of cellular pellets taken with and without induction confirm induction-dependent GST-E6 expression, with protein observed with addition of IPTG (lane 8) and absent in the non-induced culture (lane 9).

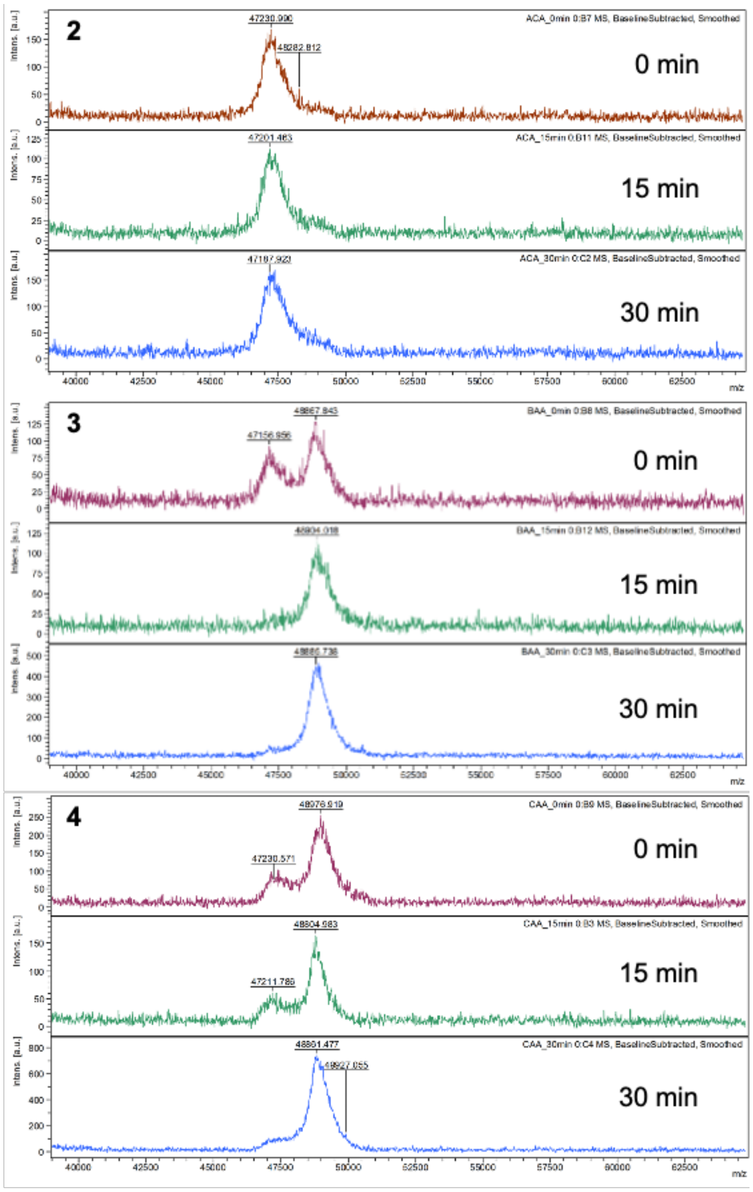

Supporting Figure S3. MALDI-MS spectrum of GST-E6 covalent labeling time course with peptides **2**-**4**. GST-E6 was incubated with 1.25 eq of each peptide at 37 °C for the indicated time.

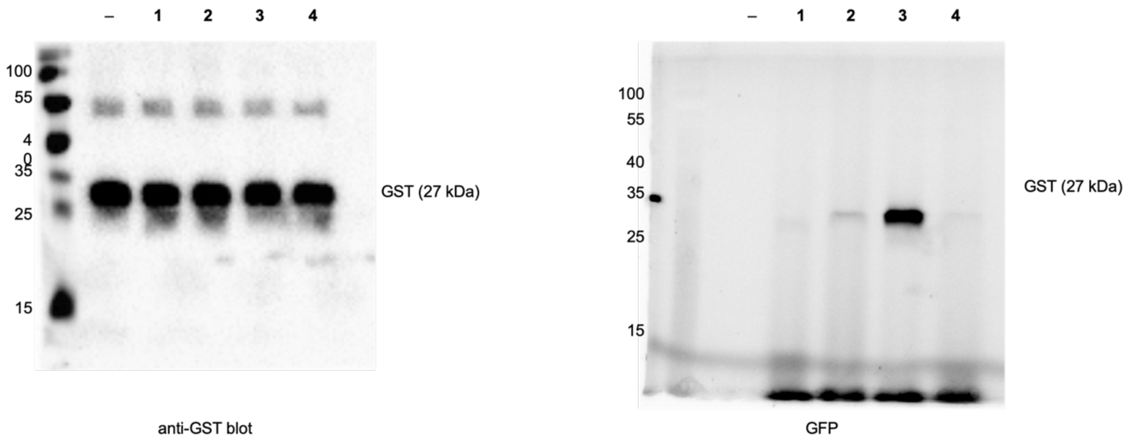

Supporting Figure S4. Selectivity of covalent peptides **2**-**4**. Recombinant GST (0.063 mg/mL, 2.33 μM) was incubated with 1.25 eq of either wild-type peptide **1**, covalent peptides **2**-**4**, or the vehicle control for 30 min at 37 °C. In-gel fluorescence (right) was used to observe the extent of off-target labeling and an anti-GST blot was used for the loading control.

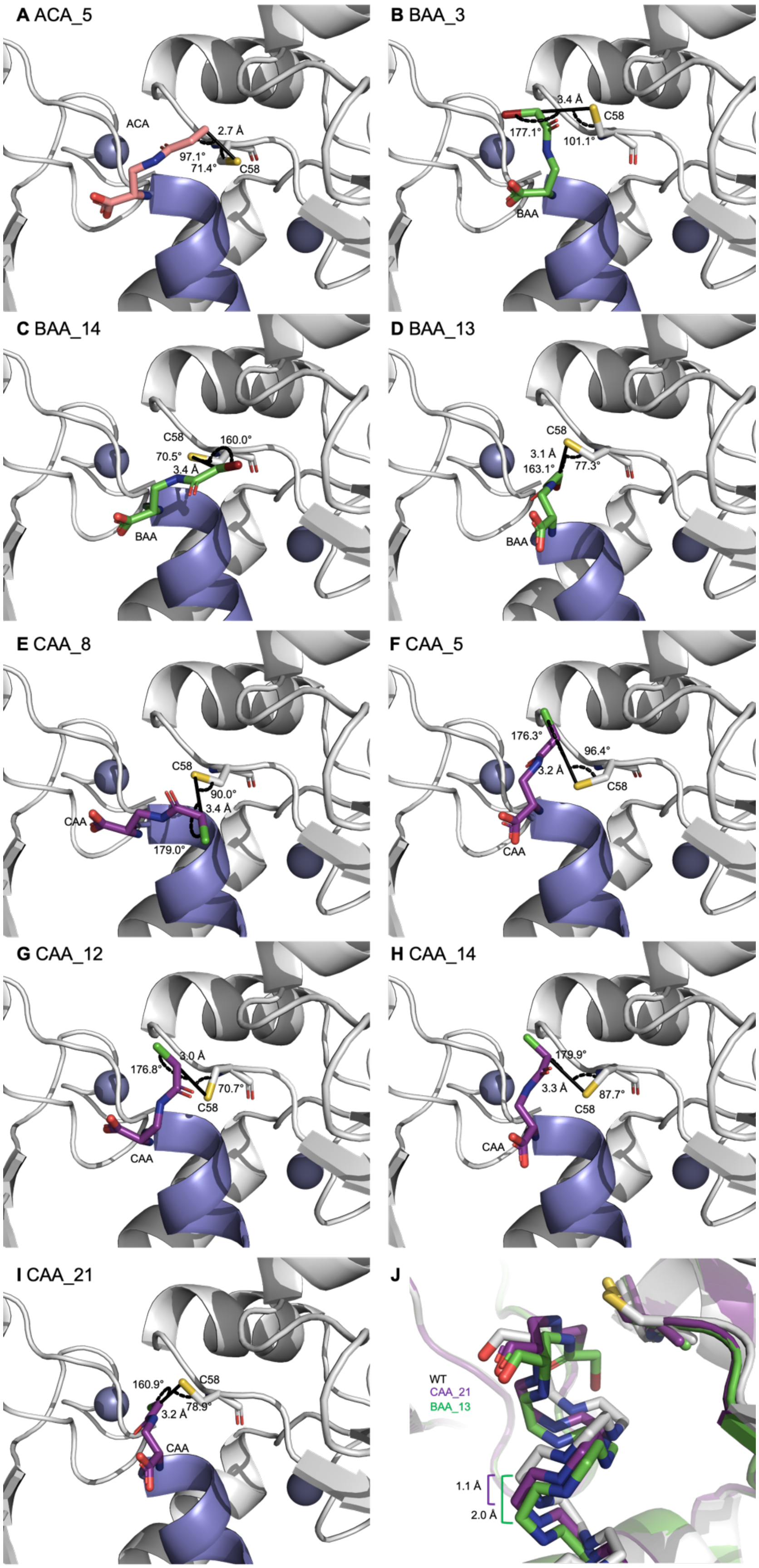

Supporting Figure S5. Electrophilic warhead rotamers that satisfy attack geometry. A) is the acrylamide rotamer. B), C), and D) are example bromoacetamide rotamers, with D) BAA_13 being energetically unfavorable. E), F), G), H), and I) are example chloroacetamde rotamers, with I) CAA_21 being energetically unfavorable. J) is the superimposition of rotamers CAA_21 (purple) and BAA_13 (green) with the wildtype (white) to show peptide displacement that may contribute to unfavorability.

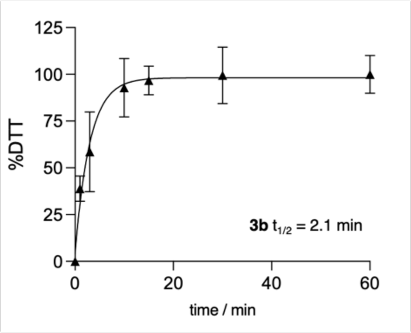

Supporting Figure S6 Determination of DTT reactivity of covalent warhead **3b** by HPLC. The percent DTT adduct formed, as determined by the measured product peak area, is plotted versus reaction time. Data represent mean normalized peak area for DTT adduct (±SD, n = 3) and nonlinear fit was applied using GraphPad Prism

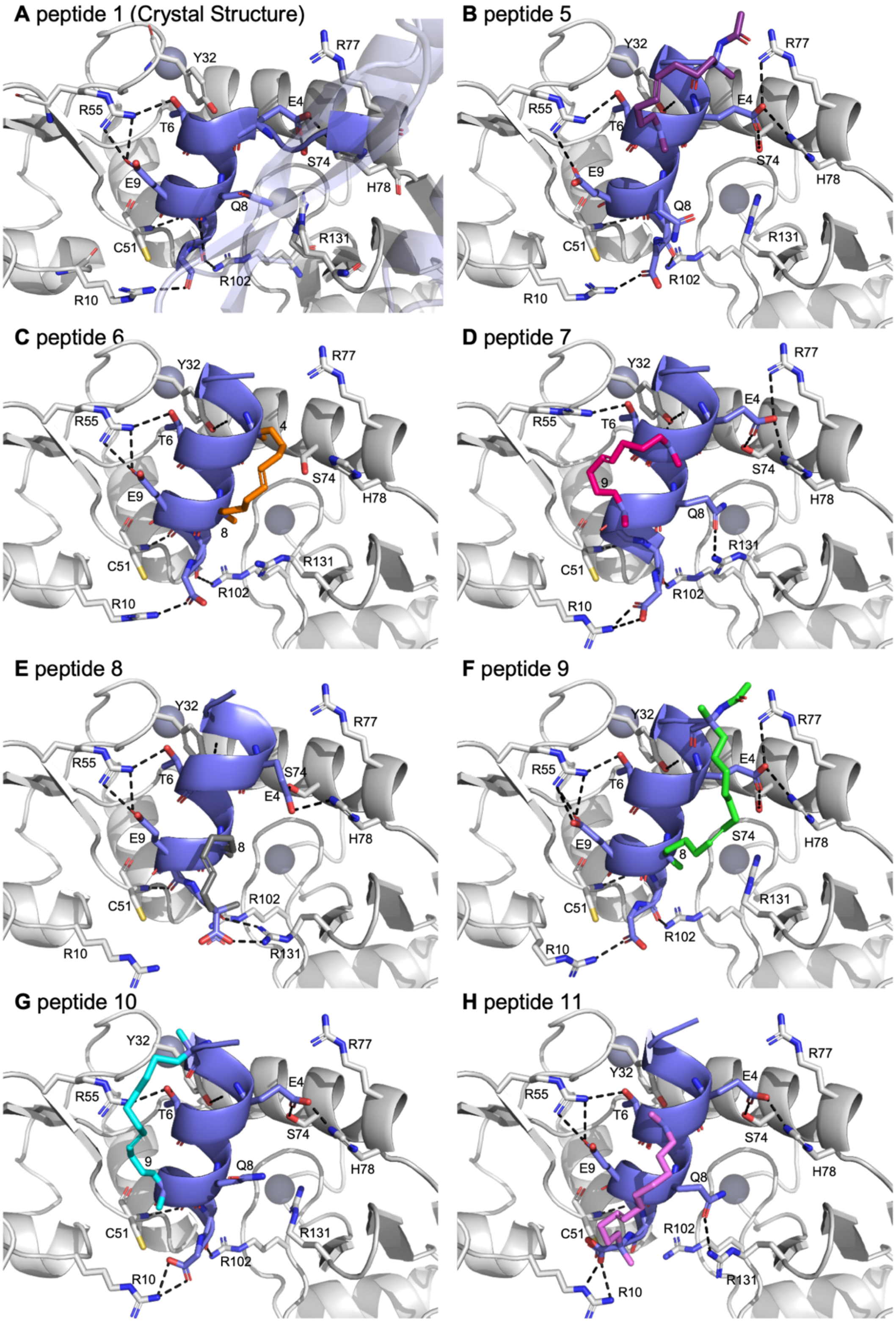

Supporting Figure S7. Models of Bound Complexes of A) Peptide 1 from PDB 4XR8 crystal structure, B) Peptide 5, C) Peptide 6, D) Peptide 7, E) Peptide 8, F) Peptide 9, G) Peptide 10, H) Peptide 11. Dashed black lines refer to polar contacts between E4, T6, Q8, E9 of the peptides and R10, Y32, C51, R55, S74, R77, H78, R102, and R131 of E6, shown in sticks.

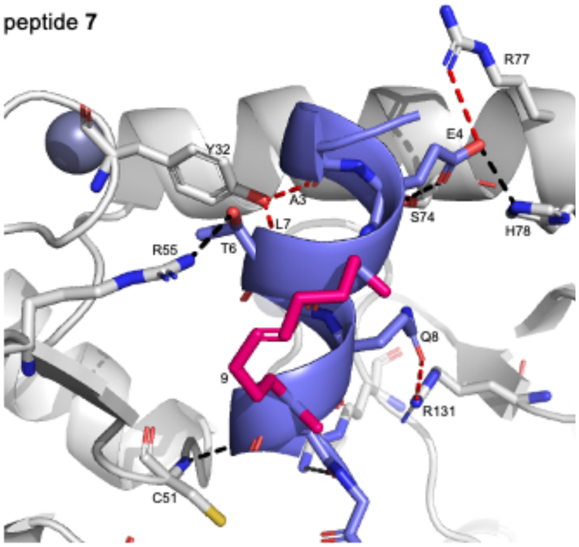

Supporting Figure S8. Additional Peptide 7-E6 Interactions. Dashed black lines refer to hydrogen bonds between Peptide 7 and E6. Red dashed lines refer to the additional interactions (E4-R77,Q8-R131, A3-Y32, and L7-Y32) not made in the crystal structure for peptide 1.

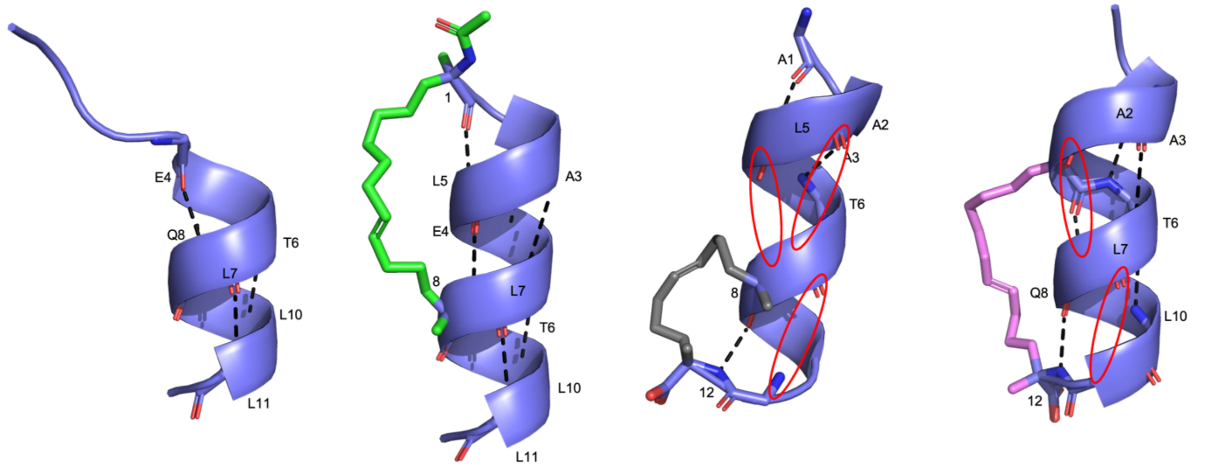

Supporting Figure S9. Distorted C-terminal Stapled Peptides. A) Peptide 1 from PDB 4XR8 and B) Peptide 9 as references. C) Peptide 8 and D) Peptide 11 staple the C-terminus Dashed black lines refer to i,i+4 helical hydrogen bonds. Red circles highlight lost helical hydrogen bonds.

Table S1. Mass spectrometry characterization.

Peptide	Sequence	% Purity	Exact Mass Calculated	Observed m/z ([M+H]$^+$; *[M+Na]$^+$)
**1**	FAM-βAla-AAAELTLQELLG-$CONH_2$	99	1655.77	1655.83
**2**	FAM-βAla-AAAELTLQELL(ACA)-$CONH_2$	99	1738.81	1739.34
**2b**	Ac-W-βAla-AAAELTLQELL(ACA)-$CONH_2$	98	1608.85	1608.94
**3**	FAM-βAla-AAAELTLQELL(BAA)-$CONH_2$	100	1806.72	1806.31
**3b**	Ac-W-βAla-AAAELTLQELL(BAA)-$CONH_2$	99	1676.76	1698.84*
**4**	FAM-βAla-AAAELTLQELL(CAA)-$CONH_2$	100	1760.77	1761.91
**4b**	Ac-W-βAla-AAAELTLQELL(CAA)-$CONH_2$	100	1630.81	1631.01
**5**	FAM-βAla-(S5)AAE(S5)TLQELLG-$CONH_2$	100	1721.82	1745.16*
**6**	FAM-βAla-AAA(S5)LTL(S5)ELLG-$CONH_2$	100	1649.90	1649.03
**7**	FAM-βAla-AAAE(S5)TLQ(S5)LLG-$CONH_2$	100	1663.81	1664.06
**8**	FAM-βAla-AAAELTL(S5)ELL(S5)-$CONH_2$	97	1720.86	1721.12
**9**	FAM-βAla-(R8)AAELTL(S5)ELLG-$CONH_2$	97	1748.89	1749.18
**10**	FAM-βAla-A(R8)AELTLQ(S5)LLG-$CONH_2$	96	1747.90	1771.43*
**11**	FAM-βAla-AAAE(R8)TLQELL(S5)-$CONH_2$	98	1777.88	1778.24
**12**	FAM-βAla-(R8)AAELTL(S5)ELL(CAA)-$CONH_2$	95	1854.89	1854.29

Abbreviations: FAM = fluorescein amide; βAla = beta-alanine; ACA = acrylamide; BAA = bromoacetamide; CAA = chloroacetamide; S5 = (*S*)-2-(4-pentenyl)Ala-OH; R8 = (*R*)-2-(7-octenyl)Ala-OH; Ac = acetyl.

Table S2. ACA rotamers with attack geometries. Green highlights acceptable geometric values (distances within 0.7A, angles within 30°), yellow highlights acceptable rotamers

ACA Rotamers	Distance between Cys S and ACA C	Angle between Cys S and ACA C=C	Angle between Cys C-S and ACA C
1	3.33	60.56	65.88
2	3.74	65.23	68.12
3	4.26	70.34	63.74
4	3.17	74.51	69.36
5	2.72	97.14	71.39
6	3.68	62.78	103.48
7	2.85	69.93	105.06
8	3.48	62.39	106.26

Table S3. BAA rotamers with attack geometries. Green highlights acceptable geometric values (distances within 0.7A, angles within 30°), ellow highlights acceptable rotamers

BAA Rotamers	Distance between Cys S and BAA C	Angle between Cys S and BAA C-Br	Angle between Cys C-S and BAA C
1	2.66	72.14	92.86
2	2.83	70.69	69.67
3	3.41	177.14	101.15
4	4.82	10.86	82.96
5	2.94	67.69	78.13
6	2.65	72.72	82.75
7	5.05	18.15	83.95
8	3.77	152.39	62.62
9	4.81	8.64	86.42
10	4.87	0.00	89.39
11	3.01	154.66	80.79
12	3.02	148.43	75.43
13	3.09	163.10	77.30
14	3.40	160.01	70.53
15	2.79	70.45	96.94
16	2.77	71.36	97.65
17	2.65	73.09	78.77
18	4.97	15.41	80.35
19	4.04	156.91	72.73
20	4.86	0.00	77.24
21	2.79	69.94	93.18
22	2.97	156.88	77.58
23	2.64	72.57	89.19
24	2.85	70.03	70.43

Table S4. CAA rotamers with attack geometries. Green highlights acceptable geometric values (distances within 0.7A, angles within 30°), yellow highlights acceptable rotamers.

CAA Rotamers	Distance between Cys S and CAA C	Angle between Cys S and CAA C-Cl	Angle between Cys C-S and CAA C
1	3.06	157.75	68.81
2	2.80	67.80	78.80
3	4.69	3.44	70.45
4	3.10	156.03	87.39
5	3.21	176.28	96.45
6	4.57	5.23	78.36
7	4.07	154.93	63.37
8	3.42	179.02	89.97
9	4.70	3.99	73.83
10	4.49	13.96	83.82
11	2.92	65.72	73.53
12	2.99	176.75	70.68
13	4.64	4.70	84.61
14	3.32	179.90	87.71
15	3.60	177.44	70.39
16	4.64	2.11	90.86
17	4.66	2.69	71.97
18	2.81	68.38	99.92
19	3.77	153.90	64.99
20	4.69	144.98	77.36
21	3.19	160.90	78.93
22	4.55	6.44	93.34
23	3.27	151.49	86.27
24	2.74	69.36	99.92
